# Model-Based Heterogeneous Data Fusion for Reliable Force Estimation in Dynamic Structures under Uncertainties

**DOI:** 10.3390/s17112656

**Published:** 2017-11-17

**Authors:** Babak Khodabandeloo, Dyan Melvin, Hongki Jo

**Affiliations:** Civil Engineering and Engineering Mechanics, The University of Arizona, Tucson, AZ 85721, USA; Khodabandeloo.babak@gmail.com (B.K.); dyanmelvin@email.arizona.edu (D.M.)

**Keywords:** force estimation, heterogeneous sensor network, Kalman filtering, multi-metric measurements, structural dynamics

## Abstract

Direct measurements of external forces acting on a structure are infeasible in many cases. The Augmented Kalman Filter (AKF) has several attractive features that can be utilized to solve the inverse problem of identifying applied forces, as it requires the dynamic model and the measured responses of structure at only a few locations. But, the AKF intrinsically suffers from numerical instabilities when accelerations, which are the most common response measurements in structural dynamics, are the only measured responses. Although displacement measurements can be used to overcome the instability issue, the absolute displacement measurements are challenging and expensive for full-scale dynamic structures. In this paper, a reliable model-based data fusion approach to reconstruct dynamic forces applied to structures using heterogeneous structural measurements (i.e., strains and accelerations) in combination with AKF is investigated. The way of incorporating multi-sensor measurements in the AKF is formulated. Then the formulation is implemented and validated through numerical examples considering possible uncertainties in numerical modeling and sensor measurement. A planar truss example was chosen to clearly explain the formulation, while the method and formulation are applicable to other structures as well.

## 1. Introduction

Many engineering structures are subjected to various natural and man-made dynamic loads, including wind, earthquake, traffic, machine vibrations, and tidal loads. The structures can be damaged severely when the applied loads are stronger than the structural capacities. Even for seemingly small level loads, their continuing and long-term application may cause gradual degradation of the structural performances over time, such as fatigue problems, so identifying the time histories of applied forces to structures can significantly improve their design and effectively protect them against damaging loading events. However, direct measurements of applied forces are impossible in practice or very difficult and costly for dynamic structures. Even if structural responses (e.g., acceleration, velocity, displacement, or strain) are measured at the locations where the forces are applied, capturing the external forces from the measured responses is not possible. Even in the case that transducers are available for force measurements, when the structure is large such as long span bridges, towers, and skyscrapers, or when the spatial distribution of loading is complex, covering the entire structure at the locations where forces would be applied is a challenging task. Therefore, indirect methods for force identification have been investigated to overcome such limitations [1,2,3].

Input force identification is the procedure to determine the loads applied to the structure using its measured responses. In other words, the goal is to find the inputs to the system using the known or measured outputs. Theoretically, having the frequency response functions (FRF) matrix together with the measured responses, it is possible to estimate the applied forces by inverting the FRF matrix. However, a unique solution is usually not available, because of the rank deficiency in the FRF matrix. The FRF-based input estimation is an ill-posed problem in general, so the presence of noise and small deviations will cause significant errors that are far from reality [4,5,6,7]. To overcome this issue for FRF-based methods, additional information, e.g., spatial distribution of loads, must be needed to have a unique solution [8,9]. Classical time and frequency domain methods for input identification also suffer from the requirement of needing an exact model, which is not possible in practice [10].

A variety of the load reconstruction approaches have been developed for specific force patterns; including impulsive load estimation for composite structures [6,11], harmonic force estimation in rotating machinery [12], and moving load identification in bridge and railway systems [13,14]. A sum of weighted accelerations technique (SWAT) is a well-known method for reconstructing impulsive loads from measured responses. In this method, measured accelerations are scaled by effective weights, which are the coefficients of equivalent mass at each acceleration location, to estimate the applied loading. This method is only suitable for systems with free boundary conditions [15,16]. For impact estimation of nonlinear structures, artificial neural network-based methods are known to improve the quality of the load estimation [17,18]. Such methods, unlike those based on convolution relation, can construct the nonlinear relationship between inputs and outputs. However, the mathematical model and algorithm used in this method has to be trained carefully by applying known forces to the structure; which is a critical limitation when applying it to large structures that have complex load patterns and distributions. A Bayesian inference-based regularization method has been proposed to identify the excitation forces [19]; they have estimated the low frequency components with good accuracy but the results for higher frequencies are not given.

In contrast to the force identification methods in frequency domain methods based on the fast Fourier transform, time domain methods were developed [20,21,22]. These methods in general consist of two main steps. First, using an operation matrix, the sequences of inputs are mapped to the outputs. In the second step, ill-posed inverse problem is solved with a regularization method. However when the size of operation matrix increases the force identification becomes highly difficult to solve. To overcome the problem of large operation matrix, sequential deconvolution input reconstruction (SDR) method was proposed [23]. Extensive parametric study of sequential deconvolution for input reconstruction was conducted [24] and it was concluded that the method can identify the inputs accurately in a moderately noisy environment.

Techniques based of the Kalman Filtering (KF) method have proven to be effective in identification of different types of loadings [25]. As a recursive linear state estimator, the Kalman Filter can provide statistically optimal estimates of the state, even with uncertainties in modeling and measurements, in the sense that the error covariance matrix is minimized. It has application in many areas, including navigation, object tracking, economics, signal processing, etc. [26]. The Kalman Filter was initially developed for linear systems. But if states are described by nonlinear equations or the observation relation is nonlinear, the extended Kalman Filter approaches have been explored [27,28,29]. To accommodate the non-gaussian noise and nonlinear dynamical system, Ensemble Kalman Filter (EnKF), which can be considered as a variant of interacting particle systems or particle filtering, has been developed using non-parametric approach [30]. However, in certain cases EnKF has poor performance [31,32]. For example, EnKF has difficulties to handle highly non-Gaussian posterior pdfs [33].

The Kalman filtering variants have been used for response identification at unmeasured locations [34,35,36], parameter identification, and damage detection [29]. A Kalman Filter approach in conjunction with a recursive least-square algorithm has been developed for force excitation estimations [6,25]. But this method requires data vectors that contain displacement measurements at all degree of freedoms which is not possible in most of the practical applications. Deconvolution Kalman Filter (DKF) is recently introduced and estimates the input forces using an augmented state space system and Kalman filtering [37]. Augmented state space system is formed from combination of state space matrices from an auto regressive moving average (ARMA) and the mechanical system response modeling. Kalman filter-based approach is used to estimate external forces and torques for a serial-chain robotic manipulator [38]. In this case, dynamic model of the robot and available motor signals such as current, angles, and speed are used and there is no need for additional sensing. In lead-through programming (LTP), which is a fast approach for teaching a trajectory by physical interaction with robot, it is possible to use a sensor-less method based on Kalman filter using the generalized momentum formulation [39]. The quality of estimated torque relies on dynamic model of robot and the friction model.

Another variant of force identification using Kalman filter is to incorporate the unknown forces into state vectors and estimate the unknown forces as part of the states; this method is called Augmented Kalman Filter (AKF) [40]. In the original approach to use the AKF, acceleration responses are used in the measurement update of the Kalman Filter for force estimation. Stability issues of the AKF method have been investigated when accelerations are the only measured responses [41]. Because the error covariance matrix of AKF has the simple form of Riccati equations, it is discussed based on analytical arguments that estimations based solely on acceleration measurement are inherently unstable. To overcome the instability of input estimation from measured accelerations, dummy measurement method is suggested [41]. But if there are slowly varying low-frequency forces, the method will not be able to trace them. The other source of numerical instabilities in force estimation is when there are more sensors than the order of reduced-order dynamic model; reduced order models are frequently used since limited number of vibration modes dominate the response of a structure subjected to dynamic loading [37]. Furthermore, joint input-state estimation was applied in structural dynamics response and input estimation when accelerations are measured [42] and was further extended to account for unknown stochastic excitations [43]. To resolves the numerical issues related to the rank deficiency and un-observability of the AKF, dual Kalman filter approach for joint input-state estimation was proposed [44] and experimentally validated [45]. The mentioned limitations of AKF are solved through successive structure of dual Kalman filter.

Acceleration responses are usually the easiest and cheapest to measure and have been widely used in structural system identification and response and force estimations. However, their performance is not satisfactory in the low frequency range [46]. On the other hand, strain measurements work perfectly at low frequencies and show direct relations to stress, hence failure and fatigue [47]. Force identification relying solely on acceleration response measurements suffer from deficiencies such as instability, inaccuracy, and even possibly misleading results. Incorporation of displacement measurement is one remedy for the problem but it is expensive and complicated.

**Figure 1:**
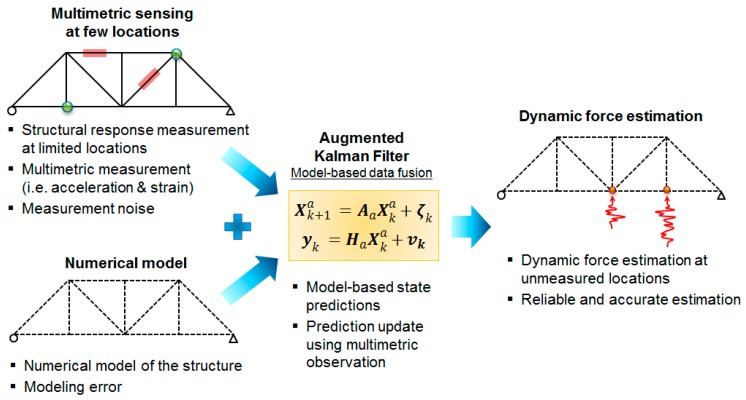
Schematic of proposed model-based heterogeneous data fusion for reliable force estimation.

In this paper, a multi-metric approach is investigated to improve the stability and accuracy of the force estimation using the AKF method. It is shown how the stability issue of the AKF can be addressed and force estimation accuracy in both low- and high-frequency range can be enhanced by combined use of multi-metric measurements, i.e., strain and acceleration responses measured at limited locations of the structure, in the measurement update stage of the Kalman filter. The efficacy of the proposed method is numerically validated using a planar truss bridge structure model. The force estimation performances of the multi-metric method are compared with the applied reference loads under various modeling and loading conditions, considering nonzero-mean loads, and measurement noises. The proposed method is schematically shown in Figure 1.

## 2. Augmented Kalman Filter A

As a recursive linear state estimator, the Kalman filter provides statistically optimal estimates of the states in a way that minimizes the mean of the squared error, assuming the system behaves linearly and potential process noise and measurement noise are zero-mean Gaussian stochastic process. The states are predicted using a system (i.e., numerical model of a structure) and the predictions are updated using observations in a minimum-variance unbiased sense. This section describes the conventional Kalman filter for response estimation and its extended approach, i.e., augmented Kalman filter (AKF), for estimating both structural responses and input forces [40]. Furthermore, the stability issue of the AKF is discussed.

### 2.1. State Space Model

Dynamic behavior of a linear mechanical system is described by a second order differential equation of motion:

(1) $$M\ddot{u}\left(t\right)+C\dot{u}\left(t\right)+Ku\left(t\right)=S_{f}f\left(t\right),$$

where $M_{n\times n}$, $C_{n\times n}$, and $K_{n\times n}$ are mass, damping, and stiffness matrices and $S_{f_{n\times n_{f}}}$ is a force selection matrix. “n” is the number of degrees of freedom and “$n_{f}\leq n$” is the number of degrees of freedom which the force is applied to. The state space formulation of the above equation of motion in continuous domain is [48]:

(2) $$\dot{x}\left(t\right)=A_{c}x\left(t\right)+B_{c}f\left(t\right),$$

where the subscript “*c*” stands for the continuous case:

$$x\left(t\right)=\left\{\begin{array}{c} u\left(t\right)\\ \dot{u}\left(t\right) \end{array}\right\};\;A_{c}=\left[\begin{array}{cc} 0 & I\\ -M^{-1}K & -M^{-1}C \end{array}\right];\;B_{c}=\left[\begin{array}{c} 0\\ M^{-1}S_{f} \end{array}\right],$$

in the above equations $x_{2n\times 1}$ is the state vector. A set of equations, in addition to those describing the dynamics of the system, defined to describe the measured or observed values in term of the system states:

(3) $$y\left(t\right)=H_{c}x\left(t\right)+D_{c}f\left(t\right),$$

the state Equation (2) together with the measurement Equation (3) form the continuous state space model of the system.

The discrete time form of the state space model is defined as:

(4) $$x_{k+1}=Ax_{k}+Bf_{k},$$

(5) $$y_{k}=Hx_{k}+Df_{k},$$

where:

(6) $$A=e^{A_{c}\Delta t},$$

(7) $$B=\int_{0}^{\Delta t}e^{A_{c}\tau }d\tau B_{c}=A_{c}{}^{-1}\left(A-I\right)B_{c}$$

(8) $$H=\;H_{c},\;D=\;D_{c}.$$

The Kalman filter estimates the states in a recursive optimal manner from the state space formulation considering presence of uncertainties, either process errors ($w_{k}$) or measurement noises ($v_{k}$):

(9) $$x_{k+1}=Ax_{k}+Bf_{k}+w_{k}$$

(10) $$y_{k}=Hx_{k}+Df_{k}+v_{k}$$

### 2.2. Kalman Filter

Kalman filter has two main steps, i.e., time update and measurement update equations [27]:

Time update:

(11) $${\hat{x}}_{k+1|k}=A{\hat{x}}_{k|k}+Bf_{k-1}\;P_{k+1|k}=AP_{k|k}A^{T}+Q,$$

Measurement update:

(12) $$K_{k|k}=P_{k|k-1}H^{T}{\left(HP_{k|k-1}H^{T}+R\right)}^{-1}\;{\hat{x}}_{k|k}={\hat{x}}_{k|k-1}+K_{k|k}\left(y_{k|k}-H{\hat{x}}_{k|k-1}\right)\;P_{k|k}=\left(I-K_{k|k}H\right)P_{k|k-1},$$

where, Q and R are process (modeling) error and measurement noise covariance matrices, which are assumed to be known, constant, and independent of time and represented by $E\left\{w_{i}w_{j}^{T}\right\}=Q\delta_{ij}$ and $E\left\{v_{i}v_{j}^{T}\right\}=R\delta_{ij}$, respectively; where $\delta_{ij}$ is the Kronecker delta. For given covariance matrices $Q_{c}$ and $R_{c}$ for continuous system, the discrete Q and R can be obtained as below:

(13) $$Q=\int_{0}^{\Delta t}e^{A_{c}\tau }Q_{c}e^{A_{c}{}^{T}\tau }d\tau \;R=\;R_{c}/\Delta t$$

and their order of magnitude is determined by the order of magnitude of state vector and signal to noise ratio of sensors [40]. For more detail on the terms used in Kalman filter, see [27].

### 2.3. Augmented Kalman Filter (AKF)

Conventional formulation of the Kalman filter described above in (11) and (12) can provide optimized estimates of the structural responses as the states (i.e., displacements and velocities) with given information of input forces (f). However, the input forces are rarely known in reality, so additional methods are needed to estimate the input forces [6,25].

The Augmented Kalman Filter (AKF) approaches use the augmented state vector that includes the input force, and then estimates structural responses as well as the input forces together as the part of the states. In order to build the full system equations for the AKF, an input force model should be required. A random walk model is a general approach for the input force model, the state equation for the random walk model in continuous domain is defined:

(14) $$\dot{f}=0+\eta ,$$

where η is the input force model noise on the derivative of the force parameter, implying that the force derivative or force increment is a completely random process.

Its discrete version is known as a Martingale process as below:

(15) $$f_{k+1}=f_{k}+\eta_{k}$$

The original state vector is then extended to include the input force. Augmented state equation in continuous time form is:

(16) $$\left\{\begin{array}{c} \dot{x}\left(t\right)\\ \dot{f}\left(t\right) \end{array}\right\}=A_{ac}\left\{\begin{array}{c} x\left(t\right)\\ f\left(t\right) \end{array}\right\}+\left\{\begin{array}{c} w\\ \eta \end{array}\right\}$$

(17) $$A_{ac}=\left[\begin{array}{cc} A_{c} & B_{c}\\ 0 & 0 \end{array}\right]$$

In the above equation, $A_{ac}$, is system matrix for augmented formulation in continuous form. Observation equation is:

(18) $$y\;=H_{ac}\left\{\begin{array}{c} x\left(t\right)\\ f\left(t\right) \end{array}\right\}+v$$

(19) $$H_{ac}=\left[H\;D\right]$$

In discrete form, the augmented state vector and the state equation are:

(20) $$X_{k}^{a}={\left\{\begin{array}{c} X_{k}\\ f_{k} \end{array}\right\}}_{(n_{s}+n_{p})\times 1}$$

In the above equation $n_{s}$ and $n_{p}$ are number of states and inputs, respectively:

(21) $$X_{k+1}^{a}\;=A_{a}X_{k}^{a}+\zeta_{k}\;A_{a}=\left[\begin{array}{cc} A & B\\ 0 & I \end{array}\right]$$

Observation equation becomes:

(22) $$y_{k}\;=H_{a}X_{k}^{a}+v_{k}$$

(23) $$H_{a}=\left[H\;D\right]$$

matrices H and D are given in Equation (30).

Then the time and measurement update equations in the KF method become [40]:

Measurement update:

(24) $$L_{k}=P_{k|k-1}H_{a}^{T}{\left(H_{a}P_{k|k-1}H_{a}^{T}+R\;\right)}^{-1}\;{\hat{X}}_{k|k}^{a}={\hat{X}}_{k|k-1}^{a}+L_{k}\left(y_{k}-H_{a}{\hat{X}}_{k|k-1}^{a}\right)\;P_{k|k}=P_{k|k-1}-L_{k}H_{a}P_{k|k-1}$$

Time update:

(25) $${\hat{X}}_{k+1|k}^{a}=A_{a}{\hat{X}}_{k|k}^{a}\;P_{k+1|k}=A_{a}P_{k|k}A_{a}^{T}+Q_{a}$$

In augmented Kalman filtering, since the force vector is included in state vector, the modeling error covariance matrix Q, together with regularization matrix (S) which is the covariance matrix of input noise, η, form the augmented covariance matrix $Q_{a}$:

(26) $$Q_{a}=\left[\begin{array}{cc} Q & 0\\ 0 & S \end{array}\right]$$

To elaborate the observation (measurement) matrix $H_{ac}$ in augmented state space form, shown in the Equation (22), the following general relation between the measurement and structural responses is considered:

(27) $$y\left(t\right)=S_{a}\ddot{u}\left(t\right)+S_{v}\dot{u}\left(t\right)+S_{d}u\left(t\right)$$

$S_{a}$, $S_{v}$, and $S_{d}$ are “$n_{d}\times n$” selection matrices corresponding to acceleration, velocity, and displacement state, respectively. “$n_{d}$” is the number of measurements and “n” is the number of degrees of freedoms.

We have:

(28) $$\ddot{u}\left(t\right)=-M^{-1}C\;\dot{u}\left(t\right)-M^{-1}Ku\left(t\right)+M^{-1}S_{f}f\left(t\right)\;y\left(t\right)=S_{a}\left\{-M^{-1}C\dot{u}\left(t\right)-M^{-1}Ku\left(t\right)+M^{-1}S_{f}f\left(t\right)\right\}+S_{v}\dot{u}\left(t\right)+S_{d}u\left(t\right)\;y\left(t\right)=\left(S_{v}-S_{a}M^{-1}C\right)\dot{u}\left(t\right)+\left(S_{d}-S_{a}M^{-1}K\right)u\left(t\right)+S_{a}M^{-1}S_{f}f\left(t\right)\;\Rightarrow H=H_{c}=\left[S_{d}-S_{a}M^{-1}K\;,\;S_{v}-S_{a}M^{-1}C\right],\;D=D_{c}=S_{a}M^{-1}S_{f}$$

When strain is measured, the strains can be formulated as the linear combination of displacement states, then the general relation Equation (27) can be rewritten as:

(29) $$y\left(t\right)=S_{a}\ddot{u}\left(t\right)+S_{v}\dot{u}\left(t\right)+S_{d}u\left(t\right)+S_{s}u\left(t\right)$$

where $S_{s}$ is the strain selection matrix. Therefore, if strain measurements are considered, then (28) becomes:

(30) $$H=H_{c}=\left[S_{s}+S_{d}-S_{a}M^{-1}K\;,\;S_{v}-S_{a}M^{-1}C\right],\;D=D_{c}=S_{a}M^{-1}S_{f}$$

### 2.4. AKF Update via Multi-Metric Observation

Multi-metric approaches combining specialized metrics have tremendous potential to enhance the quality of the obtained information, providing a comprehensive way to take the respective advantages and to overcome the weakness of such single-metric methods [49,50]. Civil structures are exposed to both low- and high-frequency force excitations; therefore, the Multi-metric approach investigated here can improve the AKF accuracy for estimating broadband force excitations.

In addition, the Multi-metric method can contribute to solving the stability issue of the AKF. Naets et al., (2015) analytically pointed out the stability problem of the AKF using the Popov-Belevitch-Hautus (PBH) criterion. Particularly, when acceleration measurements are only used in the measurement update, the AKF intrinsically suffers from the instability issue [41].

Based on the PBH criterion, a system is detectable if and only if the PBH matrix (given below) has full column rank for all the eigenvalues, s, or the undetectable modes have an eigenvalue with a negative real part, i.e., are stable [41]:

(31) $$PBH=\left[\begin{array}{c} sI-A_{ac}\\ H_{ac} \end{array}\right]$$

In the above equation, to test the detectability, it suffices to check the rank of matrix for the eigenvalues of the augmented system which consist of eigenvalues of the dynamic system and zeros accounted for the unknown forces [31]. Expanding PBH using (17) and (19) it would be easier to investigate stability for different measurements:

(32) $$PBH=\left[\begin{array}{c} \begin{array}{ccc} sI & -I & 0\\ M^{-1}K & sI+M^{-1}C & -M^{-1}S_{f}\\ 0 & 0 & sI \end{array}\\ \begin{array}{ccc} S_{s}+S_{d}-S_{a}M^{-1}K & S_{v}-S_{a}M^{-1}C & S_{a}M^{-1}S_{f} \end{array} \end{array}\right]$$

In the case when acceleration only is measured in all DOFs, $S_{s}=S_{d}=S_{v}=0$ and $S_{a}=I$, then in the PBH matrix, the first and last columns are linearly dependent at *s* = 0, as shown in Equation (33). Therefore, the system is not observable when acceleration measurements only are used. The same problem occurs, when velocity only is measured:

(33) $$PBH=\left[\begin{array}{c} \begin{array}{ccc} 0 & -I & 0\\ M^{-1}K & M^{-1}C & -M^{-1}S_{f}\\ 0 & 0 & 0 \end{array}\\ \begin{array}{ccc} -M^{-1}K\; & -M^{-1}C & M^{-1}S_{f} \end{array} \end{array}\right]$$

Another reason to consider zero eigenvalue, i.e., *s* = 0, to check the detectability is to account for possible uncertainties available in the systems, numerical modeling, and response measurements. For real dynamic systems, the case having zero eigenvalue do not exist. But such uncertainties can result in zero eigenvalue in the observation. Thus, detectability of the system should be ensured for all eigenvalues, including zero eigenvalue.

In the case of displacement measurements available, regardless of whether full or partial displacement measurements are used, the system is detectable in the presence of system damping [41]. However, measuring absolute displacements in the field is a challenging task for full-scale civil structures.

For the case where strain measurements are used, considering the observation matrix which contains the strain selection matrix, see Equation (30), the same argument made for displacement measurements hold and the system would be detectable in the presence of damping even for partial strain measurements. When accelerations together with strains are measured, i.e., the Multi-metric observation, $S_{s}\neq 0$, $S_{d}=S_{v}=0$ and $S_{a}\neq 0$; then the PBH matrix becomes:

(34) $$PBH=\left[\begin{array}{c} \begin{array}{ccc} sI & -I & 0\\ M^{-1}K & sI+M^{-1}C & -M^{-1}S_{f}\\ 0 & 0 & sI \end{array}\\ \begin{array}{ccc} S_{s}-S_{a}M^{-1}K & -S_{a}M^{-1}C & S_{a}M^{-1}S_{f} \end{array} \end{array}\right]$$

Even if accelerations and strains for all DOFs are not measured, in presence of damping the non-observable modes would be stable and therefore detectability of the system is satisfied. Instead of using acceleration and strain measurements, one potential solution may be using acceleration measurements to estimate velocity and displacement via numerical integration. This may seem a straightforward and easy task but indeed it is not. Especially it is very difficult to handle nonzero direct current (DC) value or low frequency components even in numerical simulation (see pages 51–55 in [51]). In practice, accelerometers are not sensitive to very low frequency and they can’t measure static forces. Moreover, data acquisition (DAQ) systems have some drift and it is complicated to separate such effects from low frequency forces. Therefore, integrating acceleration numerically does not provide us with the correct estimate of velocity and displacement.

### 2.5. Strain Selection Matrix for Planar Truss

**Figure 2:**
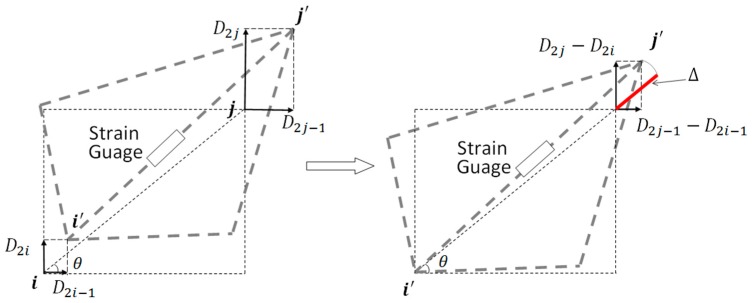
General exaggerated deformation of part of the truss structure (**Left**)—translated deformed shape to coincide the node “*i*” with its initial undeformed shape (**Right**).

Structural deformations of many civil structures can be reasonable assumed to be small, compared with the size of the structure, during their operation. Exploiting this small deflection assumption, it is possible to linearly relate the strains to the displacements (states) with high accuracy. To obtain the linear relation between strains and displacements in a planar truss structure, a general deflection of part of the truss is shown in Figure 2 (left). To see how the strains can be estimated from displacements using a linear relation, it would be simpler if the deformed shape is translated such that one of the shifted nodes coincide with its initial position, that is shifting *i’* to *i* (right in Figure 2).

Considering the geometrical relation, when the deformation is small, using Equation (35) strain is estimated from the states linearly:

(35) $$\Delta \approx \left(D_{2j-1}-D_{2i-1}\right)\cos\left(\theta \right)+\left(D_{2j}-D_{2i}\right)\sin\left(\theta \right)$$

In matrix form:

(36) $$\varepsilon =\frac{\Delta }{L}=\left[\begin{array}{cc} \begin{array}{cc} \begin{array}{cc} 0 & \cdots \end{array} & \frac{-\cos\left(\theta \right)}{L} \end{array} & \begin{array}{cc} \frac{-\sin\left(\theta \right)}{L} & \begin{array}{cc} \cdots & \begin{array}{ccc} \frac{+\cos\left(\theta \right)}{L} & \frac{+\sin\left(\theta \right)}{L} & \begin{array}{cc} \cdots & 0 \end{array} \end{array} \end{array} \end{array} \end{array}\right]\left\{\begin{array}{c} D_{1}\\ \vdots \\ \begin{array}{c} D_{2i-1}\\ D_{2i}\\ \begin{array}{c} \vdots \\ D_{2j-1}\\ \begin{array}{c} D_{2j}\\ \vdots \\ D_{2N} \end{array} \end{array} \end{array} \end{array}\right\}=S_{s}u\left(t\right)$$

**Figure 3:**
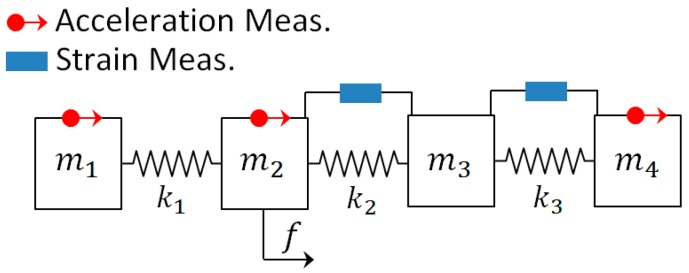
Four Degree of Freedom system with one excitation on the 2nd DOF.

To better understand the selection matrices $S_{a},\;S_{v},\;S_{d}$, $S_{s}$, and $S_{f}$ consider the simple one-dimensional lumped mass 4-DOF system shown in Figure 3:

A force (f) is applied on the 2nd mass (DOF) and the acceleration of the 1st, 2nd, and 4th DOF are measured, and the strain between the 2nd and 3rd and between 3rd and 4th DOF (masses) are measured. In the observation (measurement) vector, the first row has information of acceleration of first mass; second row has information of acceleration of second mass; third row has information of acceleration of fourth mass; fourth row has the strain between 2nd and 3rd DOF and the fourth row has the information of the strain between 3rd and 4th DOF:

$$S_{a}=\left[\begin{array}{cc} \begin{array}{cc} \begin{array}{c} 1\\ 0\\ \begin{array}{c} 0\\ 0\\ 0 \end{array} \end{array} & \begin{array}{c} 0\\ 1\\ \begin{array}{c} 0\\ 0\\ 0 \end{array} \end{array} \end{array} & \begin{array}{cc} \begin{array}{c} 0\\ 0\\ \begin{array}{c} 0\\ 0\\ 0 \end{array} \end{array} & \begin{array}{c} 0\\ 0\\ \begin{array}{c} 1\\ 0\\ 0 \end{array} \end{array} \end{array} \end{array}\;\right],\;S_{d}=S_{v}=\left[\begin{array}{cc} \begin{array}{cc} \begin{array}{c} 0\\ 0\\ \begin{array}{c} 0\\ 0\\ 0 \end{array} \end{array} & \begin{array}{c} 0\\ 0\\ \begin{array}{c} 0\\ 0\\ 0 \end{array} \end{array} \end{array} & \begin{array}{cc} \begin{array}{c} 0\\ 0\\ \begin{array}{c} 0\\ 0\\ 0 \end{array} \end{array} & \begin{array}{c} 0\\ 0\\ \begin{array}{c} 0\\ 0\\ 0 \end{array} \end{array} \end{array} \end{array}\;\right],\;S_{s}=\left[\begin{array}{cc} \begin{array}{cc} \begin{array}{c} 0\\ 0\\ \begin{array}{c} 0\\ 0\\ 0 \end{array} \end{array} & \begin{array}{c} 0\\ 0\\ \begin{array}{c} 0\\ 1\\ 0 \end{array} \end{array} \end{array} & \begin{array}{cc} \begin{array}{c} 0\\ 0\\ \begin{array}{c} 0\\ -1\\ 1 \end{array} \end{array} & \begin{array}{c} 0\\ 0\\ \begin{array}{c} 0\\ 0\\ -1 \end{array} \end{array} \end{array} \end{array}\;\right],\;S_{p}=\left[\;\begin{array}{c} \begin{array}{c} 0\\ 1 \end{array}\\ \begin{array}{c} 0\\ 0 \end{array} \end{array}\right]$$

### 2.6. Strain Selection Matrix for Planar Truss

In Finite Element Methods for solid mechanics, strain and nodal point displacements are related via the following equation:

(37) $$\left\{\varepsilon \right\}=TN\left\{u\right\}=S_{s}u\left(t\right)$$

The product of differentiation matrix operator *T*, and shape function matrix *N* is called displacement differentiation matrix. The differentiation matrix operator for different solid mechanics elements such as beams, plates and shells can be found in solid mechanics finite element methods books. For example see Chapters 2 and 3 in [52].

## 3. Simulations and Results

**Figure 4:**
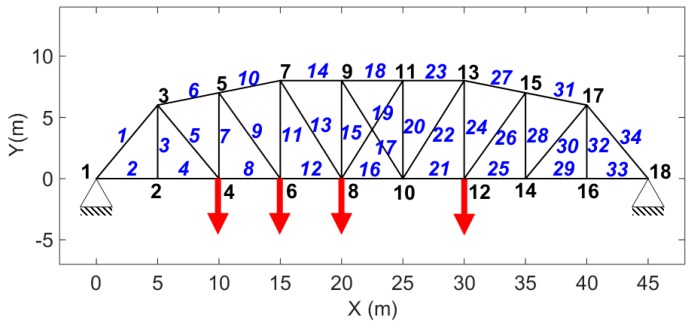
The model of the truss used for simulation—Blue italic numbers show the member number and the black ones indicate the node numbers. There are eighteen nodes and 34 members; Red bold arrows show where the simultaneous excitations are applied.

**Figure 5:**
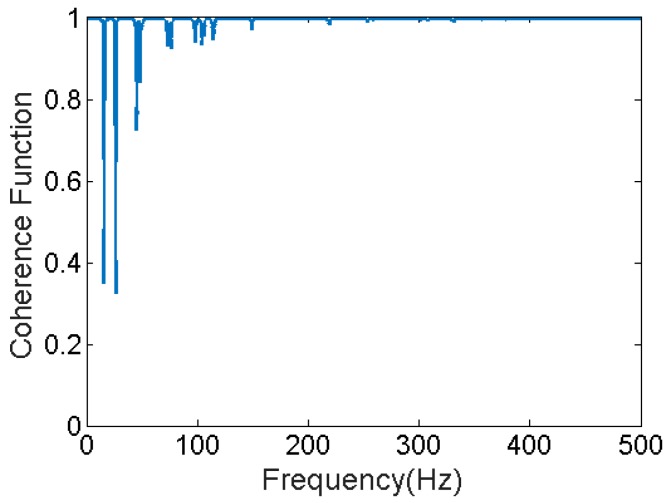
Multiple coherence function is always between zero and one. One indicates all the measured output is due to the considered inputs while zero means other factors (noises, unconsidered input, etc.) have caused the output(s).

**Figure 6:**
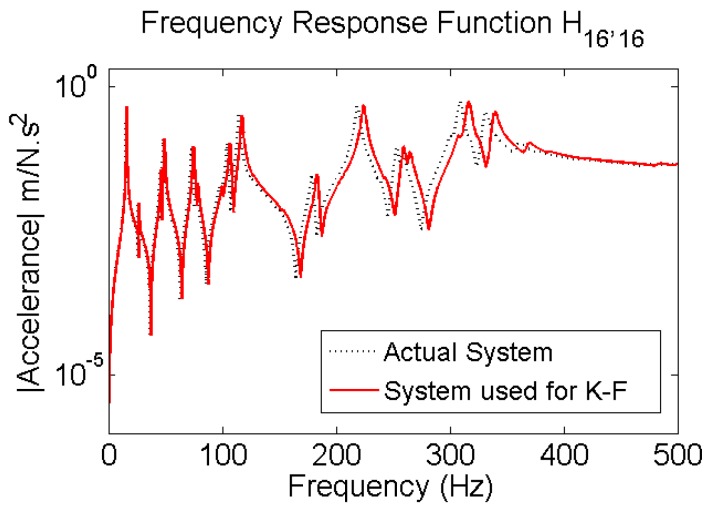
Dotted black: FRF of the system that is used for simulation of response; Solid red line: FRF of the perturbed system, to represent modeling error (5%) used for input estimation.

Responses of a planar steel truss structure model (see Figure 4) with 18 joints and 34 members are simulated using the fourth order Runge-Kuta method [53]. The time step for response simulation is 1/4096 s. Quality of the simulation is validated by calculating the multiple coherence function [54] shown in Figure 5. The coherence function is a scalar value between zero and one; in which one indicates the outputs are fully due to the inputs. At resonance, there are some drops; which are partly due to small numerical errors in solving the equation of motion and mainly due to spectral leakage and biased and random errors of spectral estimations (see [51] pp. 205–240), because finite amount of data is used to do so. The structure with its node and element numbers is depicted in Figure 4, and the properties of the elements are given in Table 1. A modal damping ratio of 0.7% was considered for all the modes of the structure. In Figure 4, red bold arrows show the applied force locations in the structure; four different excitations are simultaneously applied to the system. H16,16—Frequency Response Function (FRF) of the system between measured DOF “16” and excitation at DOF “16”, called point FRF—is shown with a dotted line in Figure 6. DOF “16” is vertical direction of joint 8. This system is used for response simulations. To have realistic situation for force estimation, the same system used for simulation is not used in the Kalman Filter. This is done because a perfect model of the structure almost never happens. A slightly different model which is 5% stiffer is used representing a modeling error (5%). The point FRF (H16,16) of this stiffer model used in K-F is shown by red solid line in Figure 6.

Several simulations are conducted with different combinations of responses, such as accelerations, displacements, and strains for estimation of the applied forces using the augmented Kalman filter method. For simulating the strain, $S_{ij}\left(t\right)$, which is the strain of the link with the end nodes “*i*” and “*j*”, the following formula is used:

$$S_{ij}\left(t\right)=\frac{\sqrt{{\left[(X_{i}+D_{2i-1})-\left(X_{j}+D_{2j-1}\right)\right]}^{2}+{\left[(Y_{i}+D_{2i})-\left(Y_{j}+D_{2j}\right)\right]}^{2}}-\sqrt{{\left(X_{i}-X_{j}\right)}^{2}+{\left(Y_{i}-Y_{j}\right)}^{2}}}{\sqrt{{\left(X_{i}-X_{j}\right)}^{2}+{\left(Y_{i}-Y_{j}\right)}^{2}}}$$

**Figure 7:**
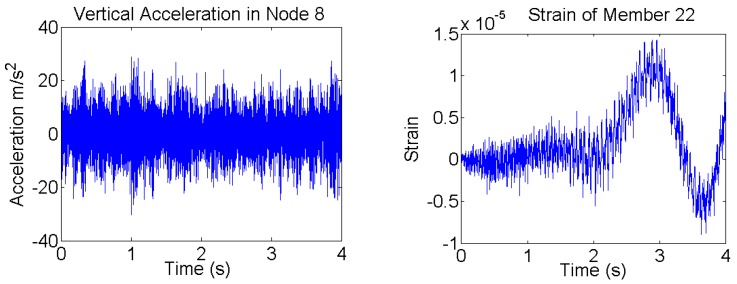
Simulated acceleration (**Left**) in vertical direction at Node “8” and strain (**Right**) in the member “22” when the forces shown in Figure 11 (dotted black) are applied to the structure simultaneously. Low frequencies (almost DC components) are seen in the strain measurements.

In Figure 7 typical accelerations and strains responses from the numerical simulations, when the system is subjected to the combination of different forces simultaneously applied to the structure, are shown. In the right of Figure 7, low-frequency variations can be clearly observed in the strain data due to very low frequency excitations applied to the system, but not in acceleration data; which are intrinsic characteristics of acceleration and strain measurements.

If there are forces with zero mean value and no (or very small) measurement noise or modeling error, it is possible to estimate the inputs using only acceleration. However, if different combinations of loading are applied and some of them have non-zero mean values then acceleration-based estimation is misleading. Different combinations of responses are simulated to use for force estimation:

Case 1

- Only acceleration measurements are used.
- System is subjected to random excitations (vertical direction at joints “4”, “8”, and “12”) and impulse applied in the vertical direction of joint “6”.
- Regarding presence of noise and modeling errors, two cases are considered: i) without modeling error and measurement noise and ii) with modeling error (5%) and measurement noise (2%).

Case 2

- Another case that only acceleration measurements are used.
- But, different forces are simultaneously applied at four nodes in vertical direction. Random (vertical direction of Joint “4”), impulsive (vertical direction of Joint “6”), random + low varying high amplitude (vertical direction of Joint “8”), noise + ramp shape (vertical direction of Joint “12”).
- Whether errors are available or not, two cases are considered: (i) without modeling error and measurement noise and (ii) with them where the results are again unstable as in case 1 when noise was considered.

Case 3

- The case that only strain measurements are used.
- Loading condition is the same as Case 2
- Modeling errors and measurement noises are considered.

Case 4

- The case that both acceleration and strain measurements are used.
- Loading condition is the same as Case 2
- Modeling errors and measurement noises are considered.

### 3.1. Case 1: Acceleration Measurements Only—Random and Impulsive Excitation

#### 3.1.1. No Modeling and Error, No Measurement Noise

**Figure 8:**
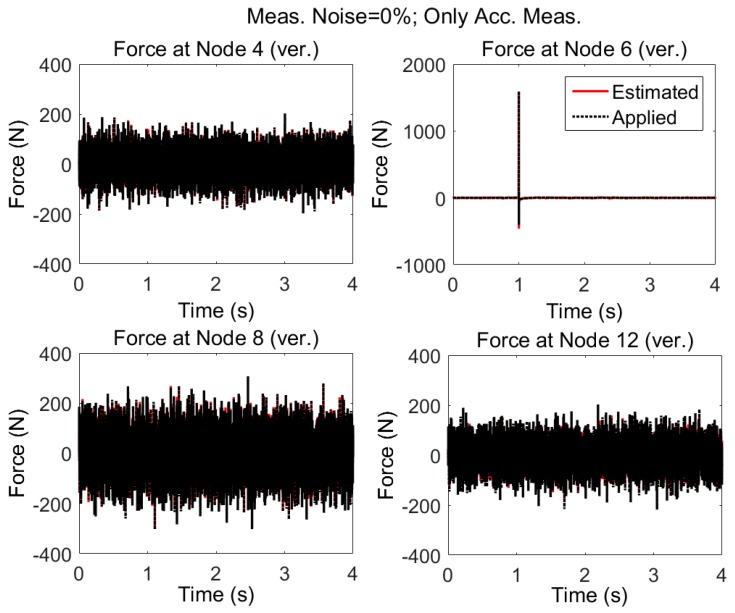
System is subjected to three uncorrelated random noise in vertical directions at nodes 4, 8, and 12 and impulse applied at node 6 (vertical). Excitations are estimated by AKF only using the accelerations; neither modeling nor measurement error is considered.

The model shown in Figure 4 is subjected to random excitations and impulse. Uncorrelated random forces are exciting the structure in the vertical direction at nodes 4, 8, and 12 and impulsive force is applied at node 6 also in the vertical direction. The applied forces are shown with dotted black lines in Figure 8. Acceleration responses are simulated and measured in vertical direction at nodes 2, 4, 5, 6, 7, 8, 9, 12, 14, 15 and in horizontal direction at nodes 4, 5, 8, and 9.

**Figure 9:**
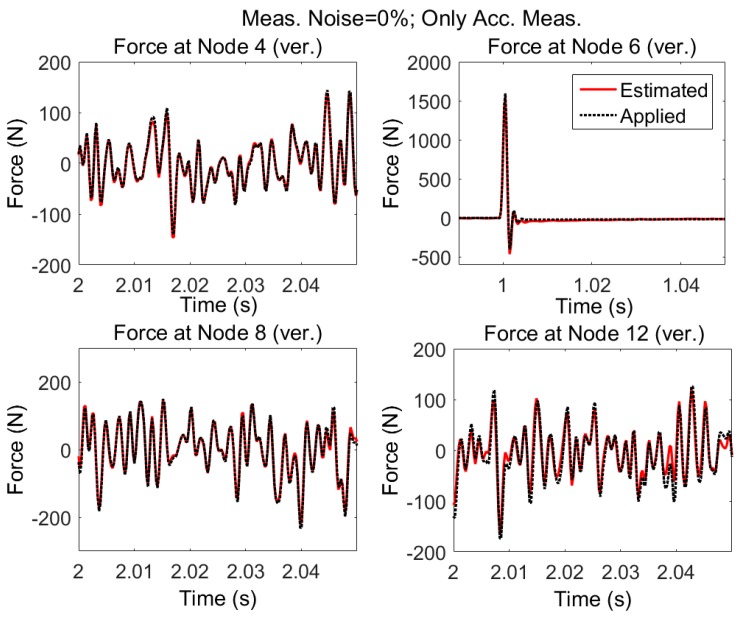
Estimated and applied forces shown for smaller time interval: detail of Figure 8.

Using the accelerations and AKF algorithm, without considering measurement noise and modeling errors, the applied forces are estimated. The results are shown in Figure 8. In Figure 9, the estimated loads are shown for a smaller time interval to see the accuracy of estimation.

It is seen that it is possible to estimate the excitation(s) using only acceleration measurements provided that:

1) there is no measurement noise and modeling error, and
2) the structure is subjected to random zero mean and impulsive forces, Then, even when forces are applied simultaneously at different locations of the structure, it is possible to estimate the input loading reliably. However, assuming no measurement errors and no discrepancy between the dynamic response of model and the real structure is too far from reality.

#### 3.1.2. Modeling Error (5%) and Measurement Noise (2%)

**Figure 10:**
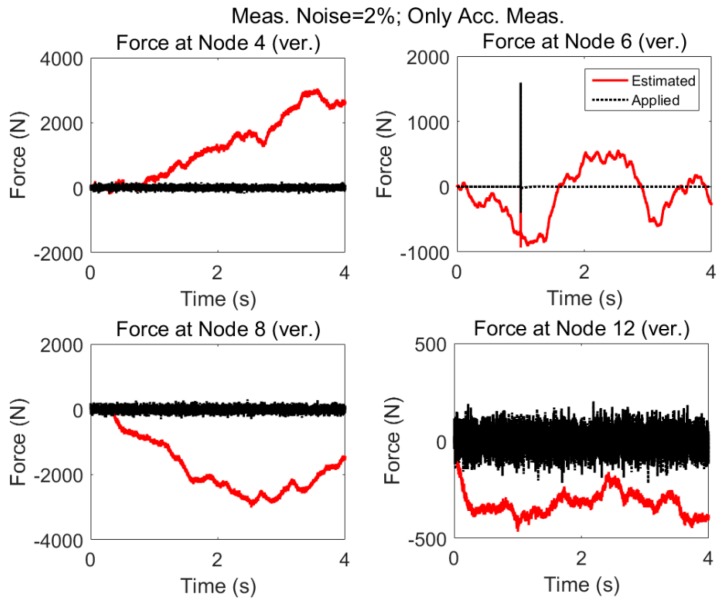
Same loading as in Figure 8, but in this case both the measurement noises (2%) and modelling errors (5%) are present.

In practice it is not possible to have an exact model, nor is it possible to have noise free measurements, so we have considered such uncertainties for force estimation. The results are shown in Figure 10. It is seen that the estimation becomes unstable; which is attributed to the fact that the AKF to use only acceleration data in the measurement update stage is not detectable as described in the Section 2.4. For undetectable systems, the states cannot be reliably estimated with the observed information. Such instability issues have been reported in other papers as well [40,41].

Therefore, when the structure is subjected to multiple zero mean random and impulsive forces, only acceleration measurements can be misleading to estimate the applied forces if there are measurement noises or modeling errors. Since such uncertainties are inevitable in almost all real-world situations, we can conclude that only acceleration measurements will end in erroneous force approximation.

### 3.2. Case 2: Acceleration Measurements Only—Presence of Low Varying and Non-Zero Mean Excitations

#### 3.2.1. No Modeling and Error, No Measurement Noise

**Figure 11:**
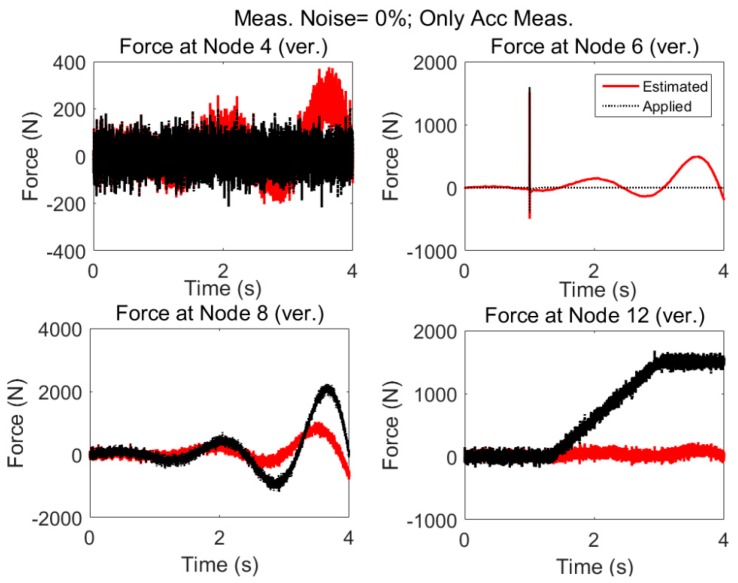
System is subjected simultaneously to four different forces where some of them have nonzero mean and low frequency and high amplitude. No error (modeling and measurement error) is added and excitation is estimated by AKF only using the accelerations. Even without added uncertainties relying solely on acceleration will fail to estimate the inputs.

The measurement configuration is same as “case 1” but the excitations are different statistically; that is in addition to random and impulsive load applied in vertical direction of nodes 4 and 6, respectively, low varying plus random excitation and ramp shape plus random excitation are applied to the nodes 8 and 12. The applied forces are shown with dotted black lines and estimated forces by the AKF are shown with red solid lines in Figure 11. Even without considering any additional uncertainties, such as measurement noise and modelling error, it is seen that only acceleration measurements are not effective in estimating low-frequency (at node 8) and nonzero-mean excitation (at node 12); also, zero mean random (at node 4) and impulsive load (at node 6). Estimations by AKF are highly biased in the presence of nonzero low varying forces. This instability in the presence of nonzero mean low varying excitations can be attributed to the rigid body modes as was discussed in the case of eigenvalues equal to zero in Section 2.4.

**Figure 12:**
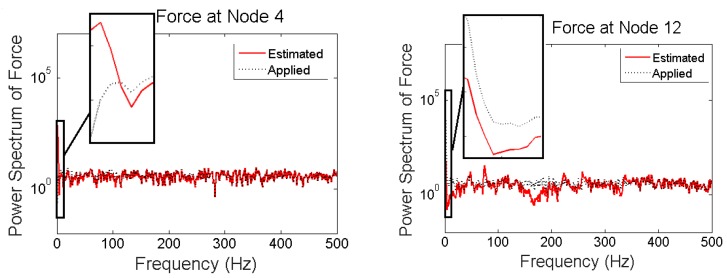
Frequency contents of applied and estimated forces at nodes 4 and 12 for “case 2”.

Power spectrums of estimated and applied forces at two locations are given in Figure 12. It is seen, even though no modeling error or measurement noises are considered, using acceleration only the low frequency components cannot be captured.

When there are nonzero low varying forces applied to the structure, it is not possible to obtain good estimates of applied forces based on only acceleration measurements even if there is no measurement noise nor modeling error. Specifically, low frequencies cannot be captured in this case.

#### 3.2.2. Modeling Error (5%) and Measurement Noise (2%)

**Figure 13:**
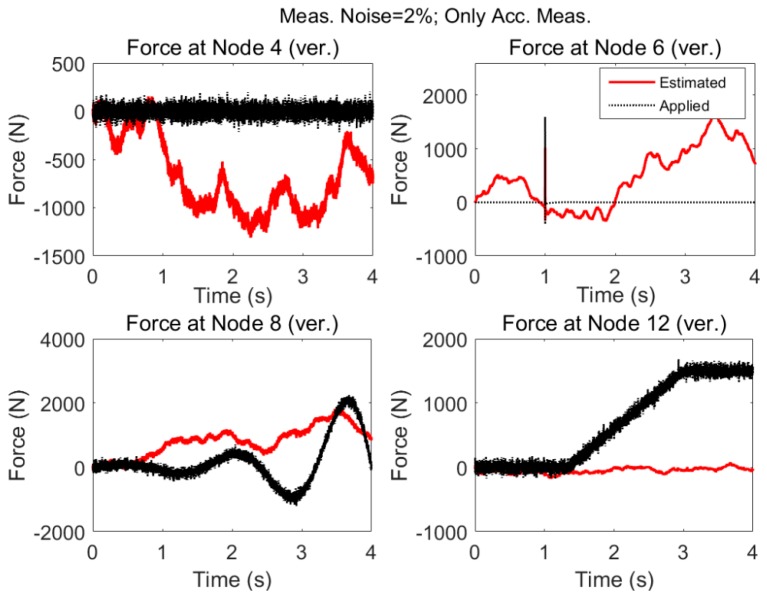
Same as Case 2 but there is modeling error (5%) and measurement noise (2%).

The results for the acceleration only case when modeling error and measurement noises are present are shown in Figure 13. As expected, the estimation fails to approximate the excitations.

As expected, in the presence of nonzero low varying forces and modeling errors and measurement noises there will be large deviations in the estimated excitations.

### 3.3. Case 3: Only Strains Are Measured—Modeling Error (5%) and Measurement Noise (2%)

**Figure 14:**
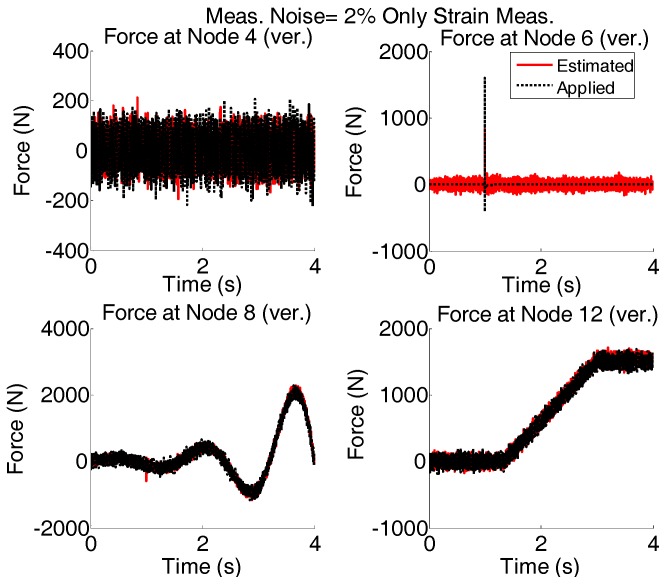
System is simultaneously subjected to four different forces shown with dotted black; Modeling (5%) and measurement error (2% of rms of the random signal) is added and excitation is estimated by AKF using the strain measurements with single acceleration measurement.

Only strain measurements would be attractive to consider; for this case of force reconstruction based only on strain measurements, Strain of members 4, 5, 9, 12, 13, 16, 21, 24, and 26 are simulated. The results of estimated forces are shown in Figure 14.

**Figure 15:**
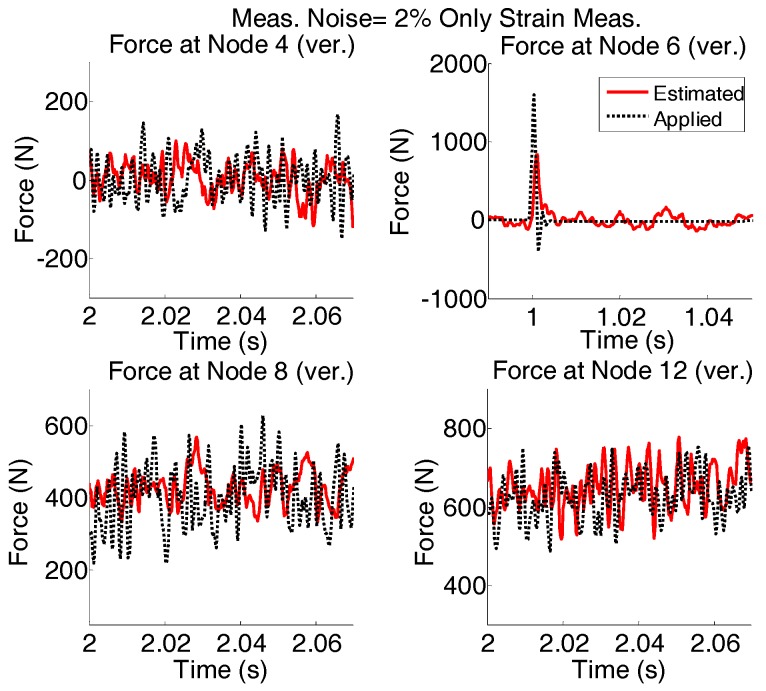
The results shown in Figure 14 are re-plotted for shorter time intervals to see the quality of estimation.

Again, the results shown in Figure 14 are plotted for shorter time interval and are given in Figure 15.

In the case of only strain measurements, it is possible to capture the low variations of the force even when both measurement noises and modeling errors are present. However, in high frequencies the variations are not captured reasonably.

### 3.4. Case 4: Strain and Acceleration—Modeling Error (5%) and Measurement Noise (2%)

Since strain measurements are simpler and more accessible than displacement measurements, a combination of acceleration and strain measurements for force estimation using AKF are investigated. The linear estimation of strains based on displacements is given in Equation (35) for planar truss structure.

**Figure 16:**
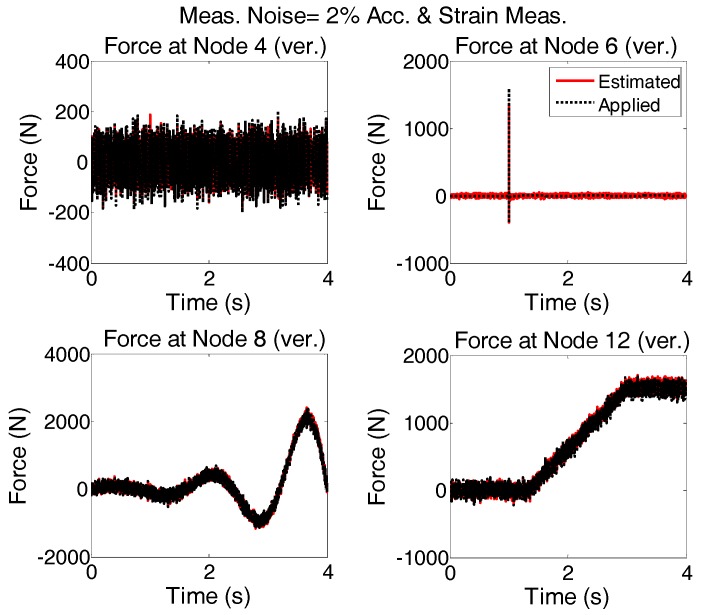
System is simultaneously subjected to four different forces shown with dotted black; Modeling and measurement error (2% of rms of the random signal) is added and excitation is estimated by AKF using the accelerations and strain measurements.

**Figure 17:**
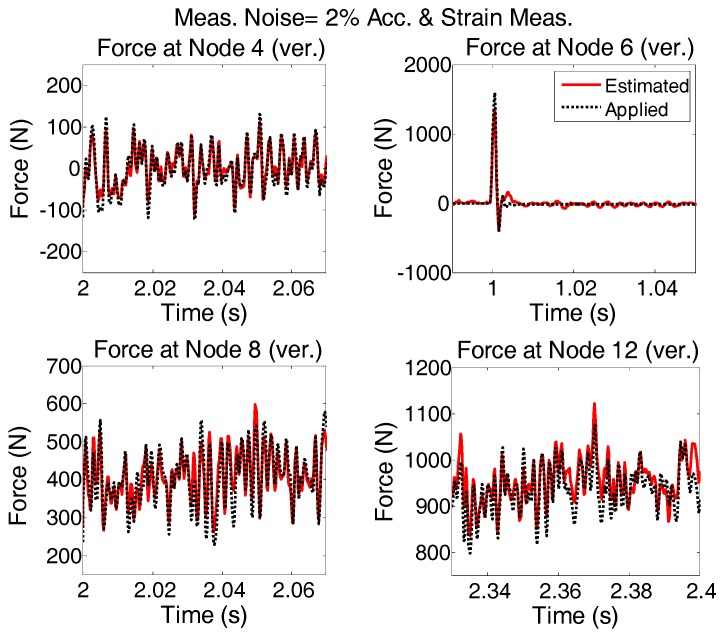
The results shown in Figure 16 are re-plotted for shorter time intervals to see how the estimation follows the applied forces in more detail.

The applied forces to the system are same as “case 2”. Modeling error and measurement noises are considered. The accelerations are measured in vertical directions at nodes 4, 6, 8, and 12 and in the horizontal direction at node 6; Strains are measured in members 5, 9, 13, and 26. To see the quality of the estimated forces, the results shown in Figure 16 are plotted for shorter time interval and are given in Figure 17.

**Figure 18:**
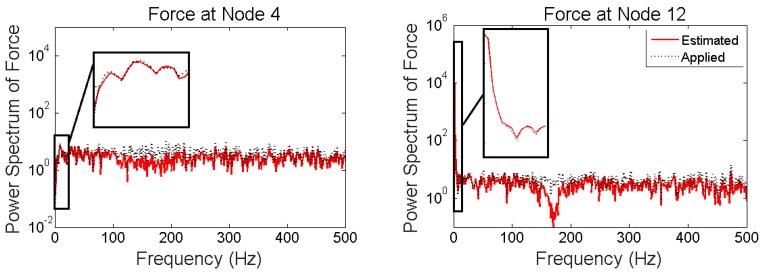
Frequency content of applied and estimated forces at Nodes 4 and 12 for “case 3”.

It is seen that in the multi-metric case, i.e., deploying strain and acceleration measurements together, the best results are achieved in low-frequency as well as high-frequency and the estimation is stable. Frequency content of estimated and applied forces at nodes 4 and 12 are shown in Figure 18.

The results of the above-mentioned cases are summarized in Table 2. For the sake of conciseness, the cases when there is no measurement noise and modelling errors were not discussed for only strain measurements (case 3) and multi-metric measurements (case 4).

### 3.5. Comparison of Different Types of Measurements

**Figure 19:**
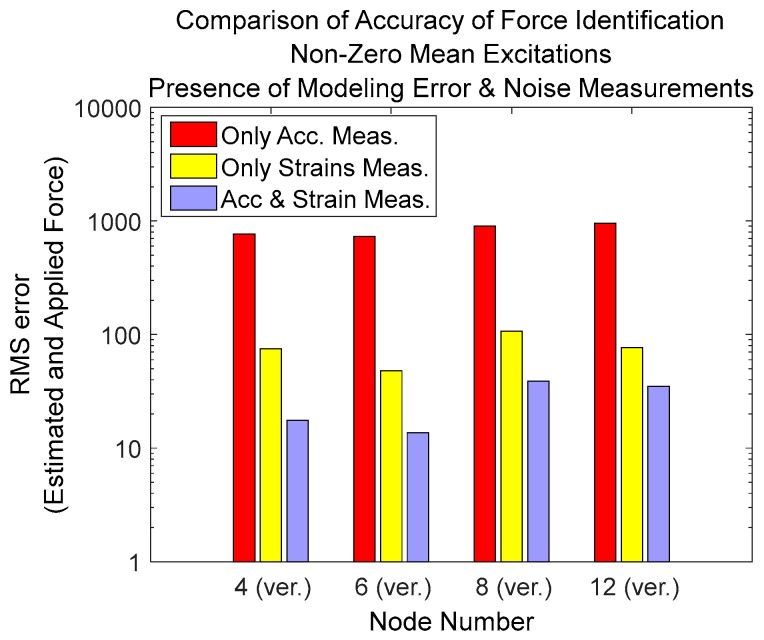
RMS error between the estimated and applied forces for different types of measurements.

For the purpose of comparing the quality of input estimation based on different types of responses or their combinations, the root mean square of the difference between estimated and applied force vectors is calculated using Equation (38) and is plotted in Figure 19:

(38) $$RMS\;error=\sqrt{\frac{1}{N_{l}}\overset{N_{l}}{\underset{i=1}{\sum }}{\left(F\left(i\right)-\hat{F}\left(i\right)\right)}^{2}}$$

in the above formula, $N_{l}$ is number of samples in the force vector, F is the applied (reference) force and $\hat{F}$ is the estimated force.

NOTE: It is also possible to use other measures to check the quality of input estimation; for example, one possible way is to compare norm of the estimated vector to the norm of reference vector divided by the norm of reference vector:

(39) $$error=100\times \frac{\left\|F\|-\|\hat{F}\right\|}{\left\|\hat{F}\right\|}$$

but in this case if low frequency estimation or the trend of estimation has good accuracy, then by increasing the amplitude of low varying excitation the RMS error will be reduced. In this case, the quality of estimation depends on the magnitude low frequency components and is misleading. Therefore, we have selected former formula in order to check the quality of estimations.

It is seen that in the case of multiple loads simultaneously applied to the system where some of them have low varying nonzero mean components, then if only accelerations are measured then the error of estimation is very high compared to the case of only strain measurements. The best result is obtained when multi-metric measurements, strain and acceleration, is used.

### 3.6. Effect of Different Configurations for Strain and Acceleration Measurements

**Figure 20:**
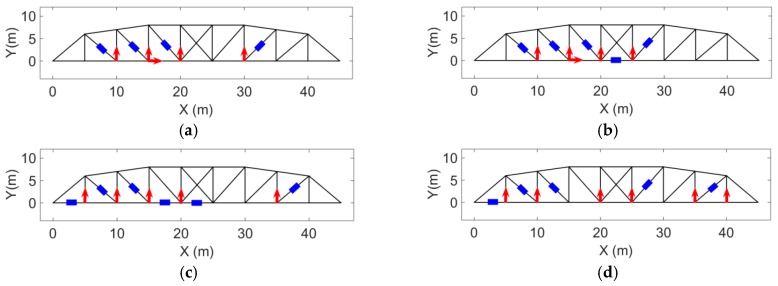
Four different strain and acceleration measurement configurations; Acceleration measurement shown by red arrow and strain measurement shown by blue rectangle. (**a**) Set 1; (**b**) Set 2; (**c**) Set 3; (**d**) Set 4.

**Figure 21:**
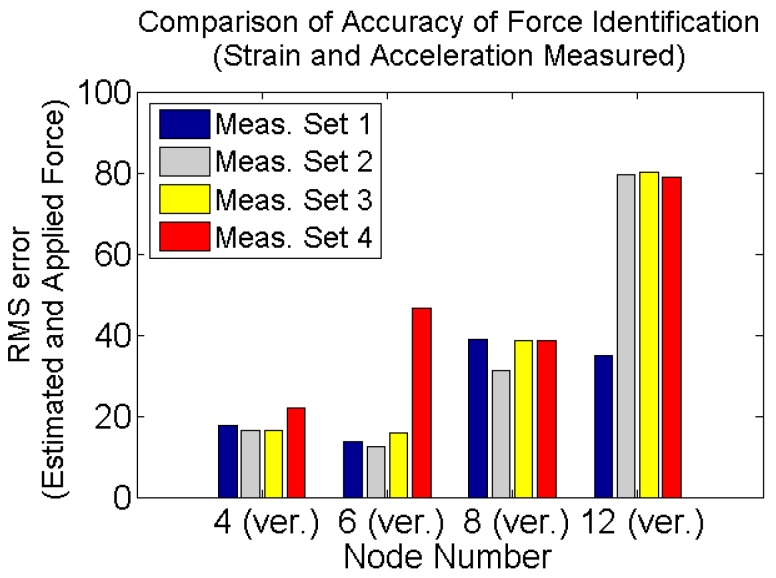
RMS error of estimated and applied forces for different strain gauges and accelerometer configurations on the truss structure, in presence of modeling error and measurement noises. Applied forces are dotted black line in Figure 11.

Four different strain and acceleration arrangements to measure the responses of the structure are considered (Figure 20). The quality of input force estimations for the applied forces described in “case 2”, when modeling errors and measurement noises are present, are compared and plotted in Figure 21.

**Figure 22:**
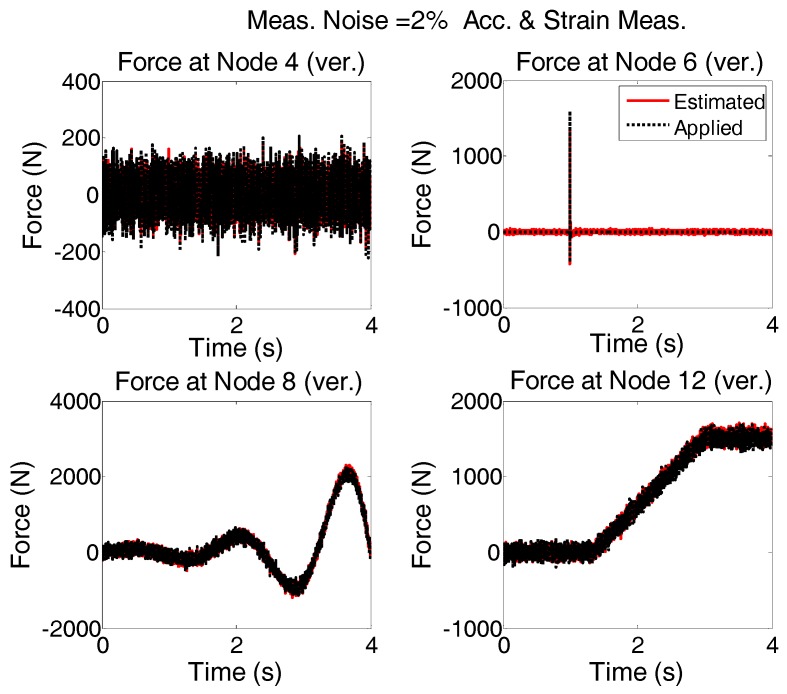
Same as Figure 16 but the measurement configuration is different. Sensor arrangement is shown in Figure 20b.

**Figure 23:**
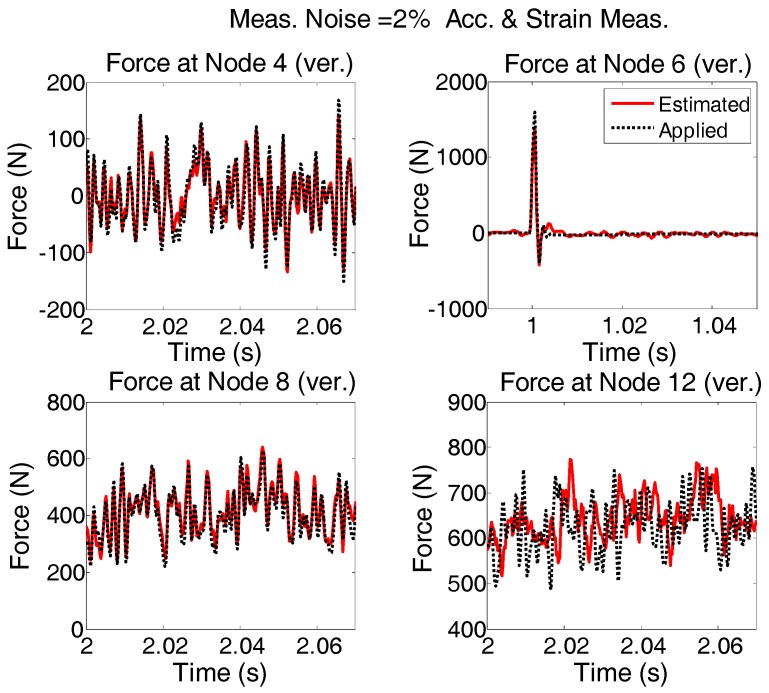
The results shown in Figure 22 are re-plotted for shorter time intervals to see the quality of estimation.

Measurement configuration (a) was used for force estimation and the results are shown in case 4. Using a different measurement configuration (b) shown in Figure 20, the same input forces applied to the structure in case 4 are estimated for the purpose of Multi-metric measurement configuration and the results are shown in Figure 22 and for smaller time interval in Figure 23.

It is seen that the results in the former sensor configuration (set “a”) for the strain and acceleration is better particularly the estimated force at Node 12. Generally, having sensors closer to the input locations increases the quality and accuracy of estimation [18,41]. It is observed that for the current formulation, using multi-metric measurements solves the stability issue. But if accurate estimation of excitation for a certain location is desired, there should be a measurement point close to that location.

## 4. Discussion and Conclusions

In this study, incorporation of strain measurements together with acceleration measurements in AKF process has been explored to provide a reliable and accurate force identification scheme for dynamic structures. Incorporating Multi-metric observations in AKF has been formulated and the method has been numerically validated using a planar truss structure simultaneously subjected to various types of forces. The proposed Multi-metric AKF approach showed significant improvements both in reliability and accuracy of dynamic force estimations under uncertain numerical modeling and measuring environment.

When only acceleration measurements, which are cheap and easy-to-obtain in practice, were used, AKF was found to be reliable just for the cases where none of the measurement noises, modeling errors, low frequency and nonzero forces are present (see Table 2). Use of displacement measurements resolves the stability issues, but are expensive and difficult to deploy in practice. On the other hand, strain measurements are easier and more practical in many cases. As a remedy to the inaccuracy and instability with the force estimation problem, AKF to use both acceleration and strain measurements was formulated. And its efficacy was validated using numerical simulation of a truss structure subjected to broad band excitations. To have more realistic simulation, measurement noises (2%) and modeling error (5%) were considered in AKF. additionally, it was observed that using only strain measurements for AKF can estimate the low-varying components of excitation with good accuracy but not for the high-frequency components. The results for different measurement configurations and for different cases were summarized in Table 2. In the future, researching the optimization of measurement configuration will be carried out and experiments on the proposed multi-metric approach for AKF will be conducted both in lab- and field-scale testbeds to further examine its performance.

## Figures and Tables

**Table 1:** Properties of the truss structure.

Elements	Young Modulus (Pa)	Cross Section Area (cm^2^)	Density Kg/m^3^
1, 6, 10, 14, 18, 23, 27, 31, 34	200 × 109	15	7800
2, 3, 4, 7, 8, 11, 12, 15, 16, 20, 21, 24, 25, 28, 29, 32, 33	200 × 109	9.75	7800
5, 9, 13, 17, 19, 22, 26, 30	200 × 109	4.75	7800

**Table 2:** Results summary for different cases. The symbols are: Yes (√), No (✕), and Not Applicable (-).

Configurations and Frequency Ranges		Random and Impulsive Excitation		Random and Impulsive Excitations + Low Varying and Non-Zero Mean Excitations	
		No Measurement Noise & No Modelling Error	2% Measurement Noise & 5% Modelling Error	No Measurement Noise & No Modelling Error	2% Measurement Noise & 5% Modelling Error
**Acc.**	Low Frequency	-	-	✕	✕
	High Frequency	√	✕	✕	✕
**strain**	Low Frequency	-	-	√	√
	High Frequency	✕	✕	✕	✕
**Acc. + strain**	Low Frequency	-	-	√	√
	High Frequency	√	√	√	√
